# Disability and sexual and reproductive health outcomes among women in Cambodia: a cross-sectional analysis of the 2021–22 demographic and health survey

**DOI:** 10.3389/frph.2026.1897583

**Published:** 2026-07-31

**Authors:** Mariko Fukao, Haruka Sakamoto, Su Myat Han, Sayaka Horiuchi, Hanako Iwashita, Samith Mey, Shuhei Nomura

**Affiliations:** 1 Department of Global Health Policy, University of Tokyo, Tokyo, Japan; 2 Graduate School of Public Health, St. Luke's International University, Tokyo, Japan; 3 School of Tropical Medicine and Global Health, Nagasaki University, Nagasaki, Japan; 4 Department of Infectious Disease Epidemiology and Dynamics, Institute of Tropical Medicine, Nagasaki University, Nagasaki, Japan; 5 Department of Epidemiology and Global Public Health, Gifu University, Gifu, Japan; 6 Department of Hygiene and Public Health, Tokyo Women's Medical University, Tokyo, Japan; 7 Phnom Penh Center for Independent Living, Phnom Penh, Cambodia; 8 Global Health Policy Lab, International Research Institute of Disaster Science (IRIDeS), Tohoku University, Sendai, Japan; 9 Department of Health Policy and Management, School of Medicine, Keio University, Tokyo, Japan; 10 Global Health Policy Lab, Graduate School of Medicine, Tohoku University, Sendai, Japan

**Keywords:** Cambodia, demographic and health survey, disability, family planning, sexual and reproductive health, unintended pregnancy, Washington group short set on functioning

## Abstract

**Background:**

Women with disabilities may face communication, autonomy-related, provider-level, and structural barriers to sexual and reproductive health (SRH) services. Population-based evidence from low- and middle-income countries remains limited, particularly by functional domain.

**Objective:**

To examine associations between disability and contraceptive knowledge, unmet need for family planning, and unintended pregnancy among women in Cambodia.

**Methods:**

We analysed data from 19,485 women aged 15–49 years who participated in the 2021–22 Cambodia Demographic and Health Survey. Disability was measured using the Washington Group Short Set and classified by severity and functional domain. Knowledge of modern contraception was assessed among all women, unmet need among currently married or cohabiting women, and unintended pregnancy among currently married or cohabiting women with a recent live birth. Survey-weighted logistic regression was used to estimate crude and adjusted odds ratios, adjusting for age, education, wealth, residence, and marital status where applicable. Additional analyses examined functional-domain associations and stratified unintended pregnancy by age, education, and wealth.

**Results:**

Overall, 16.5% of women reported at least some functional difficulty, including 1.0% with severe disability. Severe disability was associated with lower adjusted odds of knowing a modern contraceptive method (adjusted odds ratio 0.13, 95% confidence interval 0.06–0.29). Among currently married or cohabiting women, severe disability was associated with higher adjusted odds of unmet need for family planning (adjusted odds ratio 1.93, 95% confidence interval 1.06–3.49). Among married or cohabiting women with a recent live birth, mild disability was associated with higher adjusted odds of unintended pregnancy (adjusted odds ratio 1.37, 95% confidence interval 1.05–1.79); for severe disability, the confidence interval included the null (adjusted odds ratio 1.42, 95% confidence interval 0.39–5.18). Functional-domain analyses suggested lower knowledge and higher unintended pregnancy among women with communication, hearing, remembering/concentrating, or self-care difficulties. In descriptive stratified analyses, disability-related differences in unintended pregnancy were more pronounced among older women, women with less than primary education, and women in the poorest wealth quintile.

**Conclusions:**

Disability-related SRH inequities in Cambodia vary by outcome, severity, and functional domain. Family-planning services should address communication access, supported decision-making, provider competence, privacy, and socioeconomic barriers.

## Introduction

Sexual and reproductive health (SRH) is central to health, autonomy, and human rights. The Convention on the Rights of Persons with Disabilities (CRPD) affirms the right of persons with disabilities to the same range, quality, and standard of health care, including SRH services, as others (1). This commitment is reinforced by the Sustainable Development Goals (SDGs), particularly target 3.7 on universal access to SRH care and family planning (2, 3). Nevertheless, women with disabilities are often underserved by SRH systems, despite their equal right to accessible, acceptable, and high-quality SRH services (4, 5).

Barriers may occur throughout the reproductive health pathway, from inaccessible comprehensive sexuality education and family-planning information to constrained decision-making, provider assumptions, limited privacy, inaccessible facilities, and transport or cost barriers (4, 6–9). These barriers may affect not only whether women know about contraception, but also whether they can obtain acceptable methods, use them continuously, and make reproductive decisions with adequate privacy and support.

Despite growing attention to disability-inclusive health systems, population-based evidence on SRH among women with disabilities in low- and middle-income countries (LMICs) remains limited. Existing studies suggest that persons with disabilities may have lower access to modern contraception, and that family-planning knowledge, education, age, and wealth are important correlates of contraceptive use (10, 11). However, disability-related differences in reproductive health outcomes are not uniform (10, 12, 13). A multi-country Demographic and Health Survey (DHS) analysis found that women with disabilities were not consistently disadvantaged across all reproductive indicators and, in some settings, reported lower unintended pregnancy (14). These mixed findings highlight the need for country-specific analyses that examine distinct SRH outcomes separately.

Three gaps are particularly important for policy. First, much of the existing evidence is of limited use for national planning because it comes from small-scale, qualitative, geographically restricted, or otherwise non-representative samples (4, 6, 15). Second, nationally representative studies often rely on a single binary disability indicator, although barriers to SRH care may differ by functional domain (11, 14). Third, although intersectionality provides an important framework for understanding how disability may intersect with other forms of social disadvantage, few studies have examined whether disability-related differences are larger among women who are also disadvantaged by age, low education, or poverty (4, 16, 17). Addressing these gaps is essential for identifying which women are most likely to be missed by family-planning programmes and which barriers may require disability-specific responses.

Cambodia is an important setting in which to examine these questions. The country has expanded SRH and family-planning services as part of its broader health and development agenda, yet people with disabilities continue to report barriers to health care (18–21). Cambodia-specific qualitative studies have described inaccessible SRH information, constrained reproductive self-advocacy among women with disabilities, and provider-side challenges in disability-inclusive maternity care (6, 21). However, nationally representative evidence on disability-related differences in SRH remains scarce. The 2021–22 Cambodia Demographic and Health Survey (CDHS), which included the Washington Group Short Set on Functioning (WG-SS), provides an opportunity to examine these differences using internationally comparable disability measures (22–24).

We therefore used nationally representative 2021–22 CDHS data to examine associations between disability and three SRH outcomes among women aged 15–49 years: knowledge of modern contraception, unmet need for family planning, and reported unintended pregnancy. We examined these associations by disability severity and functional domain, and explored whether disability-related differences in reported unintended pregnancy varied by age, education, and household wealth. We hypothesised that associations between disability and SRH outcomes would vary by severity and functional domain, and that these associations would be more pronounced among women facing socioeconomic disadvantage.

## Materials and methods

### Study design and data source

We conducted a secondary cross-sectional analysis of the 2021–22 CDHS, a nationally representative household survey implemented by the National Institute of Statistics in collaboration with the Ministry of Health of Cambodia (24). The survey used a stratified two-stage cluster sampling design based on the sampling frame from the 2019 Cambodia General Population Census (24). In the first stage, enumeration areas were selected with probability proportional to size and stratified by urban and rural residence. In the second stage, households were systematically selected within each enumeration area. The Women's Questionnaire was administered to women aged 15–49 years who were usual residents of selected households or who had stayed in the household the night before the survey (24).

We used the CDHS 2021–22 Individual Recode and Person Recode files. Disability information was collected in the Household Questionnaire and was available in the Person Recode file. We linked the Individual Recode file to the Person Recode file using cluster number, household number, and individual line number (v001 = hv001, v002 = hv002, and v003 = hvidx). This study followed the Strengthening the Reporting of Observational Studies in Epidemiology (STROBE) guidelines for cross-sectional studies (25).

### Study population and sample

**Figure 1:**
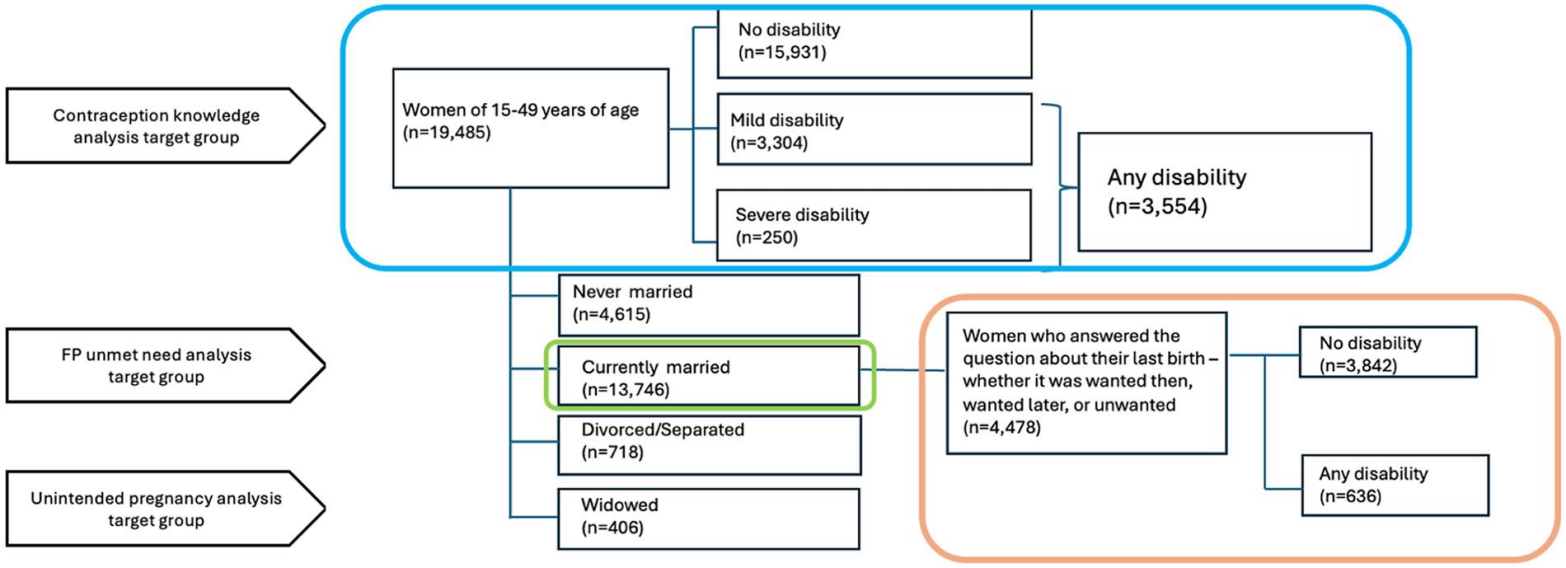
Flow of participants from the eligible Cambodia demographic and health survey 2021–22 sample to the analytic subsamples for each SRH outcome.

The primary analytic sample included 19,485 women aged 15–49 years with completed Women's Questionnaire interviews. Because each outcome was defined for a different respondent group in the CDHS, we used a separate analytic sample for each outcome. Knowledge of modern contraception was analysed among all women in the primary sample (*n* = 19,485). Unmet need for family planning was analysed among currently married or cohabiting women, consistent with DHS definitions (*n* = 13,746) (26). Unintended pregnancy was analysed among currently married or cohabiting women whose most recent live birth occurred within the five years preceding the survey, the group for whom retrospective pregnancy intention was collected (*n* = 4,478). Figure 1 shows the participant flow from the eligible survey population to each analytic sample.

### Disability assessment

Disability was assessed using the WG-SS, an internationally comparable instrument designed to identify functional difficulties in population-based surveys (22, 23). The WG-SS asks whether respondents have difficulty in six functional domains: seeing, even when wearing glasses; hearing, even when using a hearing aid; walking or climbing steps; remembering or concentrating; self-care, defined as washing all over or dressing; and communicating in their usual language. Response options are “no difficulty”, “some difficulty”, “a lot of difficulty”, and “cannot do at all”. “Don't know” and “refused” responses were rare and were treated as missing.

We operationalised disability in three ways. First, we created a three-level severity variable: no disability, defined as no difficulty in all six domains; mild disability, defined as some difficulty in at least one domain but no domain with a lot of difficulty or inability; and severe disability, defined as a lot of difficulty or inability in at least one domain. This classification retained milder functional difficulties while distinguishing the threshold commonly recommended for identifying more severe functional limitation in WG-SS analyses (22, 23). Because disability prevalence estimates depend on both measurement approach and severity threshold, we examined disability using severity categories, a binary any-disability indicator, and domain-specific indicators (27). Second, we created a binary indicator of any disability, defined as at least some difficulty in any WG-SS domain. Third, we created six domain-specific indicators, each coded as present when a respondent reported at least some difficulty in that domain. These three operationalisations were used to assess severity gradients, overall disability-related differences, and variation by type of functional difficulty.

Respondents were classified by disability status only when valid responses were available for all six WG-SS domains. Respondents with missing information in any of the six domains were coded as missing for disability status.

### Outcomes

We examined three SRH outcomes aligned with family-planning access and reproductive autonomy: knowledge of modern contraception, unmet need for family planning, and unintended pregnancy.

Knowledge of modern contraception was assessed among all women and coded as present if the respondent reported knowing at least one modern contraceptive method. Modern methods followed the standard DHS classification and included female and male sterilisation, intrauterine device, implant, injectable, pill, condom, female condom, lactational amenorrhoea method, emergency contraception, standard days method, and other modern methods (26).

Unmet need for family planning was assessed among currently married or cohabiting women using the revised DHS algorithm (26). Women were classified as having unmet need if they were fecund, not using contraception, and either wished to delay the next birth by at least two years or wished to stop childbearing.

Unintended pregnancy was assessed among currently married or cohabiting women whose most recent live birth occurred within the five years preceding the survey. The pregnancy was coded as unintended if the respondent reported that it had been wanted later or not wanted at all, and as intended if it had been wanted at the time.

### Covariates

Covariates were selected *a priori* based on previous literature on disability and SRH outcomes (4, 11, 14, 16). We included age group (15–24, 25–34, and 35–49 years), highest educational attainment (less than primary, primary completed, and secondary or higher), household wealth quintile, place of residence (urban or rural), and current marital status. Marital status was classified as never married, currently married or cohabiting, divorced or separated, and widowed. Marital status was included only in models for knowledge of modern contraception, which were estimated among all women; it was not included in models for unmet need for family planning or unintended pregnancy because those analyses were restricted to currently married or cohabiting women.

### Missing data

Missing data were rare. Disability status was missing for 11 women, who were excluded from the analytic sample. Unintended pregnancy was coded for all women with non-missing information on whether the most recent live birth was wanted then, wanted later, or not wanted at all. Knowledge of modern contraception, unmet need for family planning, age, household wealth, place of residence, and current marital status had no missing data. Educational attainment was missing for 34 of 19,485 women (0.17%), who were excluded from regression models that adjusted for education. We used complete-case analysis for adjusted regression models. Because missing data affected fewer than 1% of observations for any variable, complete-case analysis is unlikely to have introduced material bias.

### Statistical analysis

We used survey-weighted analyses to account for the stratified two-stage cluster sampling design of the CDHS. All estimates incorporated sampling weights, clustering, and stratification. The survey design was specified using women's sampling weights divided by 1,000,000, primary sampling units, and strata. For analyses restricted to specific respondent groups, such as currently married or cohabiting women, we used subpopulation estimation so that standard errors reflected the original survey design.

We first described the weighted prevalence of disability overall and across sociodemographic groups. Disability was summarised using the three-level severity classification: no disability, mild disability, and severe disability. We also described the weighted prevalence of each SRH outcome by disability severity, reporting percentages with 95% confidence intervals.

We then used survey-weighted logistic regression to estimate crude odds ratios (ORs) and adjusted odds ratios (AORs), with 95% confidence intervals (CIs), for the association between disability severity and each SRH outcome. Women without disabilities were the reference group. Adjusted models included age group, educational attainment, household wealth quintile, and place of residence. Current marital status was additionally included in the model for knowledge of modern contraception, which was estimated among all women, but not in the models for unmet need for family planning or unintended pregnancy because those analyses were restricted to currently married or cohabiting women.

To assess whether findings depended on how disability was defined, we repeated the main models using the binary any-disability indicator. We then examined functional-domain-specific associations by estimating the relationship between difficulty in each WG-SS domain and each SRH outcome. In these analyses, women reporting at least some difficulty in a given domain were compared with women reporting no difficulty in that domain. Because domain-specific analyses were exploratory, we did not adjust for multiple comparisons. This increases the risk of Type I error (false-positive findings), so these estimates were interpreted as patterns of association rather than confirmatory tests.

To explore socioeconomic patterning, we restricted analyses to women with any disability and examined sociodemographic correlates of each SRH outcome. We also estimated disability-related differences in unintended pregnancy within strata of age group, educational attainment, and household wealth quintile. These stratified estimates were interpreted descriptively rather than as formal tests of interaction because the number of women with severe disability and a recent live birth was too small to support reliable interaction estimates. All tests were two-sided, and *p* < 0.05 was considered statistically significant. Analyses were conducted using Stata 17.

### Ethics statement

This study used publicly available, de-identified data from the CDHS, obtained through the DHS Program under an approved data-use agreement. The original 2021–22 CDHS was reviewed and approved by the Cambodia National Ethics Committee for Health Research and the ICF International (ICF) Institutional Review Board (24). Written informed consent was obtained from all respondents before participation; for respondents younger than 18 years, consent was obtained from a parent or guardian and assent was obtained from the respondent (24). Because the present study involved secondary analysis of de-identified public-use data, additional institutional ethics review was not required.

## Results

### Sample characteristics and disability prevalence

The analytic sample included 19,485 women aged 15–49 years: 15,931 without disability, 3,304 with mild disability, and 250 with severe disability. Overall, 15.54% (95% CI 14.79–16.32) had mild disability and 1.00% (95% CI 0.84–1.18) had severe disability based on the WG-SS (Table 1). Disability prevalence increased with age and was higher among women with lower education, rural residence, and lower household wealth. Among women aged 35–49 years, 24.80% had mild disability and 1.75% had severe disability, compared with 6.12% and 0.40%, respectively, among women aged 15–24 years. Disability was also more common among women with less than primary education and among those in the poorest wealth quintile.

**Table 1:** Sociodemographic characteristics and crude SRH outcomes by disability severity, women aged 15–49 years, Cambodia 2021–22.

Characteristic	No disability % (95% CI) *n* = 15,931	Mild disability % (95% CI) *n* = 3,304	Severe disability % (95% CI) *n* = 250
Age group, years			
15–24	93.48 (92.60–94.26)	6.12 (5.37–6.96)	0.40 (0.24–0.67)
25–34	86.80 (85.47–88.02)	12.60 (11.38–13.92)	0.61 (0.41–0.89)
35–49	73.45 (72.08–74.79)	24.80 (23.50–26.15)	1.75 (1.41–2.17)
Education			
No/less than primary	76.95 (75.64–78.21)	21.41 (20.17–22.71)	1.64 (1.35–1.99)
Primary	87.64 (86.47–88.72)	11.86 (10.83–12.98)	0.50 (0.34–0.73)
Secondary or higher	89.36 (87.27–91.14)	9.93 (8.22–11.96)	0.71 (0.38–1.32)
Marital status			
Never married	91.08 (89.93–92.11)	7.88 (6.89–9.00)	1.03 (0.75–1.43)
Currently married/cohabiting	81.18 (80.16–82.16)	17.88 (16.93–18.88)	0.94 (0.75–1.16)
Divorced or separated	82.71 (79.16–85.76)	15.70 (12.74–19.20)	1.59 (0.86–2.92)
Widowed	70.59 (64.90–75.70)	28.00 (22.91–33.72)	1.41 (0.35–5.49)
Place of residence			
Urban	87.56 (86.33–88.69)	11.59 (10.49–12.79)	0.85 (0.61–1.17)
Rural	80.46 (79.39–81.48)	18.44 (17.43–19.48)	1.11 (0.91–1.34)
Household wealth quintile			
Poorest	76.85 (74.84–78.75)	21.49 (19.66–23.43)	1.66 (1.26–2.19)
Poorer	80.39 (78.62–82.05)	18.75 (17.13–20.48)	0.86 (0.58–1.27)
Middle	84.59 (83.12–85.95)	14.38 (13.04–15.83)	1.03 (0.70–1.52)
Richer	86.36 (84.73–87.85)	12.91 (11.44–14.53)	0.73 (0.48–1.12)
Richest	87.15 (85.64–88.53)	12.01 (10.71–13.45)	0.83 (0.50–1.37)
Sexual and reproductive health outcomes			
Knowledge of modern contraception (full sample)	98.83 (98.59–99.02)	99.22 (98.75–99.51)	90.29 (83.03–94.64)
Unmet need for FP (currently married)	11.34 (10.48–12.26)	12.01 (10.42–13.79)	18.80 (11.39–29.42)
Unintended pregnancy (married, recent live birth)	17.80 (16.11–19.63)	23.82 (19.44–28.84)	23.63 (7.98–52.47)

Column n values are unweighted sample counts. Percentages and 95% CIs are survey-weighted row percentages. Values are survey-weighted percentages with 95% confidence intervals. For sociodemographic characteristics, row percentages show the distribution of disability severity within each subgroup. SRH outcome estimates are presented for the relevant analytic sample: knowledge of modern contraception among all women, unmet need for family planning among currently married or cohabiting women, and unintended pregnancy among currently married or cohabiting women with a live birth in the preceding five years. CI, confidence interval; FP, family planning; SRH, sexual and reproductive health.

At the functional-domain level, seeing difficulty was most common, followed by difficulty remembering or concentrating (Table 2). Communication and self-care difficulties were the least common. Crude SRH outcome prevalence differed by disability severity. Knowledge of modern contraception was high among women without disability and women with mild disability, but lower among women with severe disability. Among currently married or cohabiting women, unmet need for family planning increased across disability severity categories. Among currently married or cohabiting women with a recent live birth, unintended pregnancy was more common among women with mild or severe disability than among women without disability.

**Table 2:** Prevalence and severity of functional difficulties by WG-SS domain, women aged 15–49 years, Cambodia 2021–22.

Functional domain	No difficulty % (95% CI)	Some difficulty % (95% CI)	A lot of difficulty/cannot do at all % (95% CI)
Seeing	90.27 (89.64–90.86)	9.46 (8.88–10.07)	0.27 (0.19– 0.39)
Hearing	97.41 (97.03–97.74)	2.43 (2.11– 2.80)	0.16 (0.11– 0.24)
Communicating	99.03 (98.77–99.23)	0.88 (0.68– 1.13)	0.09 (0.05– 0.18)
Remembering or concentrating	91.70 (91.11–92.25)	8.01 (7.47– 8.59)	0.29 (0.21– 0.40)
Walking or climbing steps	96.25 (95.84–96.62)	3.43 (3.07– 3.84)	0.32 (0.24– 0.42)
Self-care (washing/dressing)	99.21 (98.95–99.41)	0.68 (0.49– 0.95)	0.10 (0.06– 0.17)

Values are survey-weighted percentages with 95% confidence intervals. “At least some difficulty” includes respondents reporting “some difficulty”, “a lot of difficulty”, or “cannot do at all” in the corresponding domain. CI, confidence interval; WG-SS, Washington group short set on functioning.

### Associations between disability severity and SRH outcomes

In survey-weighted logistic regression, severe disability was strongly associated with lower knowledge of modern contraception (Table 3). Compared with women without disabilities, women with severe disabilities had lower crude odds of knowing a modern contraceptive method (OR 0.11, 95% CI 0.06–0.22), and this association remained after adjustment for age, education, household wealth, residence, and marital status (AOR 0.13, 95% CI 0.06–0.29). Mild disability was not associated with lower contraceptive knowledge after adjustment.

**Table 3:** Crude and adjusted associations between disability severity, sociodemographic characteristics, and SRH outcomes, women aged 15–49 years, Cambodia 2021–22.

	Knowledge of modern contraception (full sample)		Unmet need for FP (currently married or cohabiting)		Unintended pregnancy (married, recent live birth)	
Characteristic	OR (95% CI)	AOR (95% CI)	OR (95% CI)	AOR (95% CI)	OR (95% CI)	AOR (95% CI)
Disability status						
No disability (ref.)	1	1	1	1	1	1
Mild disability	1.50 (0.92–2.47)	0.87 (0.50–1.50)	1.06 (0.88–1.28)	1.08 (0.90–1.30)	1.44 (1.10–1.89)*	1.37 (1.05–1.79)*
Severe disability	0.11 (0.06–0.22)*	0.13 (0.06–0.29)*	1.81 (1.01–3.24)*	1.93 (1.06–3.49)*	1.43 (0.40–5.10)	1.42 (0.39–5.18)
Education						
Less than primary (ref.)	1	1	1	1	1	1
Primary	1.17 (0.83–1.63)	3.77 (2.57–5.53)*	0.85 (0.72–1.00)*	0.85 (0.71–1.01)	0.93 (0.75–1.15)	1.02 (0.80–1.30)
Secondary or higher	3.64 (1.49–8.89)*	11.66 (4.73–28.72)*	0.90 (0.70–1.15)	1.01 (0.76–1.34)	0.79 (0.58–1.06)	0.92 (0.65–1.30)
Age group, years						
15–24 (ref.)	1	1	1	1	1	1
25–34	6.41 (3.85–10.65)*	1.84 (1.08–3.14)*	0.67 (0.56–0.80)*	0.67 (0.56–0.82)*	0.81 (0.62–1.06)	0.81 (0.62–1.05)
35–49	6.26 (4.14–9.46)*	2.56 (1.57–4.16)*	0.62 (0.51–0.76)*	0.59 (0.47–0.74)*	1.49 (1.12–2.00)*	1.43 (1.05–1.93)*
Marital status						
Never married (ref.)	1	1	n/a	n/a	n/a	n/a
Currently married/cohabiting	23.06 (12.53–42.45)*	26.65 (13.82–51.42)*	n/a	n/a	n/a	n/a
Divorced or separated	8.74 (3.03–25.24)*	10.78 (3.64–31.95)*	n/a	n/a	n/a	n/a
Widowed	4.52 (1.26–16.20)*	6.04 (1.52–24.02)*	n/a	n/a	n/a	n/a
Household wealth quintile						
Poorest (ref.)	1	1	1	1	1	1
Poorer	1.11 (0.72–1.70)	1.03 (0.65–1.63)	0.99 (0.82–1.19)	1.05 (0.87–1.27)	1.28 (0.98–1.68)	1.31 (1.00–1.72)
Middle	1.61 (0.95–2.73)	1.44 (0.83–2.51)	0.92 (0.76–1.11)	1.01 (0.83–1.23)	1.21 (0.91–1.62)	1.22 (0.91–1.65)
Richer	1.68 (1.05–2.70)*	1.33 (0.76–2.32)	0.74 (0.59–0.92)*	0.86 (0.69–1.09)	0.97 (0.72–1.30)	0.94 (0.69–1.29)
Richest	2.83 (1.62–4.96)*	1.93 (0.91–4.10)	0.64 (0.48–0.86)*	0.78 (0.58–1.06)	1.10 (0.78–1.55)	1.11 (0.76–1.61)
Place of residence						
Urban (ref.)	1	1	1	1	1	1
Rural	0.76 (0.53–1.09)	0.98 (0.60–1.60)	1.37 (1.14–1.64)*	1.18 (0.99–1.40)	0.93 (0.73–1.19)	0.90 (0.69–1.16)

Odds ratios were estimated using survey-weighted logistic regression. Adjusted models included age group, educational attainment, household wealth quintile, place of residence, and, for knowledge of modern contraception only, current marital status. Women without disabilities were the reference group for disability severity. CI, confidence interval; OR, odds ratio; AOR, adjusted odds ratio; SRH, sexual and reproductive health; n/a, not applicable. *p < 0.05.

Among currently married or cohabiting women, severe disability was significantly associated with higher unmet need for family planning. Compared with women without disabilities, the adjusted odds ratio was 1.93 (95% CI 1.06–3.49) for severe disability. Mild disability was not associated with higher unmet need for family planning.

Among currently married or cohabiting women with a recent live birth, mild disability was associated with higher odds of unintended pregnancy. Women with mild disabilities had higher crude odds of unintended pregnancy than women without disabilities (OR 1.44, 95% CI 1.10–1.89), and the association remained after adjustment for age, education, household wealth, and residence (AOR 1.37, 95% CI 1.05–1.79). For severe disability, the adjusted estimate was above the null, but the confidence interval included 1 (AOR 1.42, 95% CI 0.39–5.18).

### Functional-domain-specific analyses

Associations differed across the six WG-SS functional domains (Table 4). In exploratory domain-specific analyses, communication difficulty was associated with the lowest adjusted odds of contraceptive knowledge (AOR 0.15, *p* < 0.001) and the highest adjusted odds of unintended pregnancy (AOR 4.38, *p* < 0.001). These estimates should be interpreted cautiously because some domain-specific groups were small and the analyses involved multiple comparisons.

**Table 4:** Functional-domain-specific associations with SRH outcomes, women aged 15–49 years, Cambodia 2021–22.

Functional domain (With **≥** some difficulty)	Knowledge of modern contraception			Unmet need for FP			Unintended pregnancy		
	% (95% CI)	OR (95% CI)	AOR (95% CI)	% (95% CI)	OR (95% CI)	AOR (95% CI)	% (95% CI)	OR (95% CI)	AOR (95% CI)
Seeing (*n* = 2022)	99.49 (98.99–99.74)	2.50 (1.24– 5.03)*	1.41 (0.67– 2.95)	11.92 (10.08–14.03)	1.04 (0.85– 1.28)	1.07 (0.87– 1.30)	24.60 (18.28–32.24)	1.47 (0.99– 2.17)	1.31 (0.91– 1.91)
Hearing (*n* = 561)	96.86 (94.72–98.15)	0.36 (0.20– 0.63)*	0.31 (0.16– 0.57)*	12.71 (9.16–17.38)	1.12 (0.77– 1.64)	1.09 (0.74– 1.58)	36.26 (25.35–48.80)	2.56 (1.53– 4.30)*	2.70 (1.60– 4.55)*
Communicating (*n* = 271)	90.49 (82.28–95.12)	0.11 (0.05– 0.23)*	0.15 (0.07– 0.32)*	15.30 (9.75–23.21)	1.39 (0.83– 2.34)	1.36 (0.82– 2.27)	47.61 (29.93–65.92)	4.04 (1.91– 8.55)*	4.38 (2.08– 9.21)*
Remembering/Concentrating (*n* = 1,885)	98.24 (97.22–98.89)	0.65 (0.40– 1.07)	0.42 (0.24– 0.73)*	13.25 (11.03–15.85)	1.19 (0.95– 1.50)	1.19 (0.95– 1.50)	25.83 (20.33–32.23)	1.58 (1.15– 2.17)*	1.52 (1.10– 2.11)*
Walking/climbing steps (*n* = 891)	98.71 (97.56–99.33)	0.93 (0.48– 1.79)	0.66 (0.32– 1.38)	11.99 (9.19–15.50)	1.05 (0.77– 1.42)	1.07 (0.79– 1.45)	31.83 (20.92–45.17)	2.09 (1.19– 3.66)*	1.83 (1.04– 3.22)*
Self–care (*n* = 148)	95.31 (89.90–97.89)	0.24 (0.10– 0.55)*	0.34 (0.14– 0.84)*	14.42 (8.44–23.56)	1.30 (0.70– 2.39)	1.26 (0.69– 2.30)	41.94 (19.13–68.81)	3.20 (1.06– 9.63)*	3.70 (1.31–10.45)*

For each functional domain, women reporting at least some difficulty were compared with women reporting no difficulty in that domain. Estimates were obtained using survey-weighted logistic regression. Knowledge of modern contraception was assessed among all women, unmet need for family planning among currently married or cohabiting women, and unintended pregnancy among currently married or cohabiting women with a live birth in the preceding five years. OR, odds ratio; AOR, adjusted odds ratio; SRH, sexual and reproductive health. *p < 0.05.

In addition to communication difficulty, lower odds of contraceptive knowledge were observed among women with hearing difficulty (AOR 0.31, *p* < 0.001), remembering/concentrating difficulty (AOR 0.42, *p* = 0.002), and self-care difficulty (AOR 0.34, *p* = 0.019). Seeing difficulty was associated with higher crude odds of contraceptive knowledge, but this association was attenuated after adjustment; walking difficulty was not clearly associated with knowledge.

Domain-specific associations with unmet need for family planning were weaker and less consistent. None of the six functional domains was statistically significantly associated with unmet need among currently married or cohabiting women.

For unintended pregnancy, the largest domain-specific association was observed for communication difficulty. Among currently married or cohabiting women with a recent live birth, 47.61% of women with communication difficulty reported unintended pregnancy, compared with 18.35% of women without communication difficulty; this corresponded to an OR of 4.04 (*p* < 0.001) and an AOR of 4.38 (*p* < 0.001). Higher odds of unintended pregnancy were also observed among women with hearing difficulty (OR 2.56, *p* < 0.001; AOR 2.70, *p* < 0.001), self-care difficulty (OR 3.20, *p* = 0.039; AOR 3.70, *p* = 0.014), walking difficulty (OR 2.09, *p* = 0.010; AOR 1.83, *p* = 0.035), and difficulty remembering or concentrating (OR 1.58, *p* = 0.005; AOR 1.52, *p* = 0.012). Seeing difficulty had a higher point estimate, but the association did not reach conventional statistical significance.

### Sociodemographic correlates among women with disabilities

Among women with any disability, sociodemographic correlates differed across the three SRH outcomes. Knowledge of modern contraception was nearly universal in several subgroups, resulting in sparse outcome cells and unstable estimates. Education and marital status appeared to be associated with knowledge, but the magnitude of these associations should be interpreted cautiously. Women who had completed primary education had higher adjusted odds of knowing a modern contraceptive method than women with less than primary education (AOR 11.92, 95% CI 3.78–37.59). Estimates for secondary or higher education could not be obtained because knowledge was nearly universal in that group. Compared with never-married women with disabilities, currently married or cohabiting women had substantially higher adjusted odds of knowing a modern method; similar patterns were observed for divorced or separated women and for widowed women.

Among currently married or cohabiting women with any disability, no sociodemographic characteristic was statistically significantly associated with unmet need for family planning after adjustment. Among currently married or cohabiting women with any disability and a recent live birth, age and household wealth were associated with unintended pregnancy. Women aged 35–49 years had higher adjusted odds of unintended pregnancy than women aged 15–24 years (AOR 4.49, 95% CI 1.93–10.41), whereas women in the richer wealth quintile had lower adjusted odds than women in the poorest quintile (AOR 0.43, 95% CI 0.19–0.94). Full results for women with any disability are shown in Supplementary Tables 1–S3.

### Stratified analyses of unintended pregnancy

**Figure 2:**
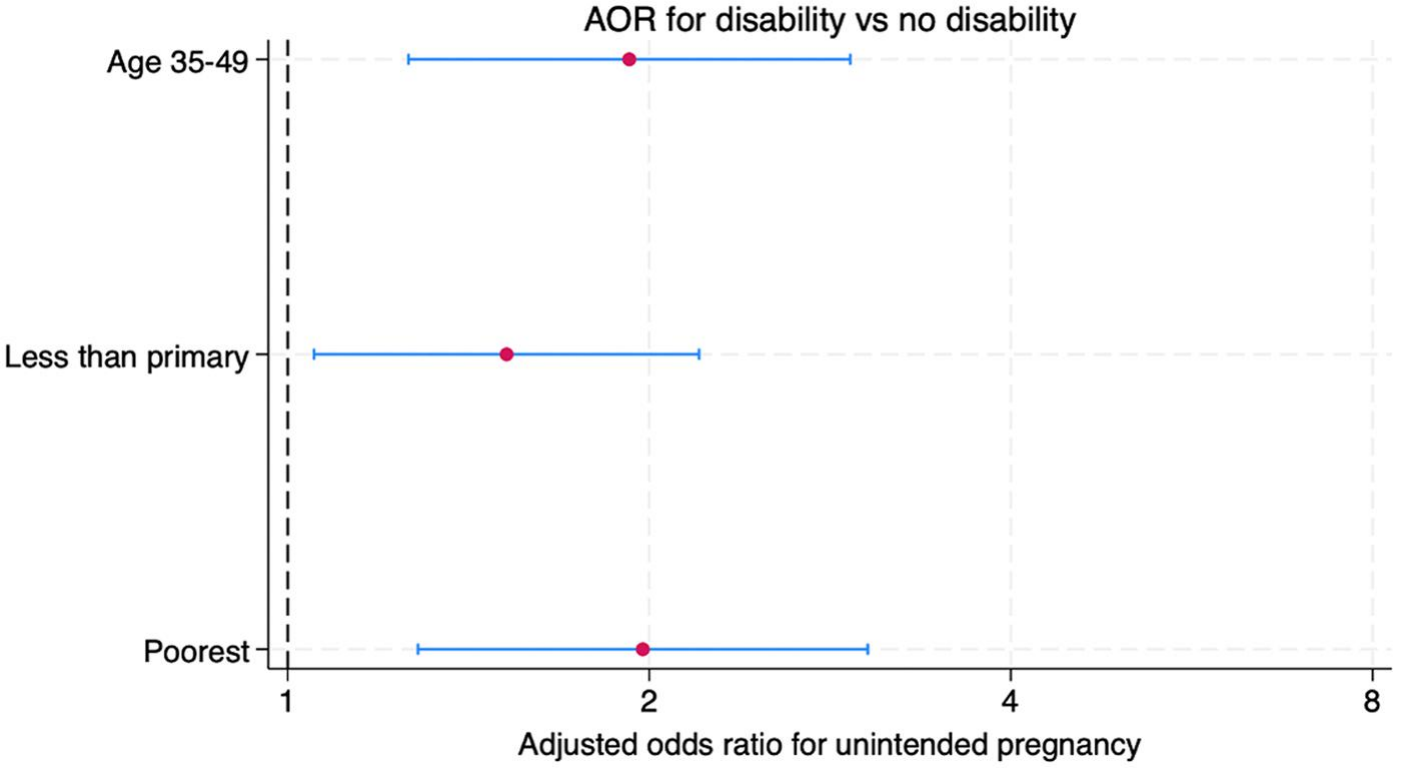
Stratified odds of unintended pregnancy among currently married or cohabiting women with vs. without disabilities, by age group, educational attainment, and household wealth quintile, Cambodia 2021–22. Adjusted odds ratios compare women with any disability with women without disability within each stratum of age group, educational attainment, and household wealth quintile. Estimates are survey-weighted and restricted to currently married or cohabiting women whose most recent live birth occurred within the five years preceding the survey. Error bars indicate 95% confidence intervals.

Descriptive stratified analyses suggested that disability-related differences in unintended pregnancy were larger among women aged 35–49 years, women with less than primary education, and women in the poorest wealth quintile (Figure 2). Because sparse data precluded reliable interaction testing, these findings should be interpreted as descriptive evidence of possible heterogeneity. Among currently married or cohabiting women aged 35–49 years with a recent live birth, women with disabilities had higher odds of unintended pregnancy than women without disabilities in the same age group (AOR 1.92, 95% CI 1.26–2.94). Similar differences were observed among women with less than primary education (AOR 1.52, 95% CI 1.05–2.20) and among women in the poorest wealth quintile (AOR 1.98, 95% CI 1.28–3.04).

## Discussion

### Principal findings

In this nationally representative analysis of Cambodian women aged 15–49 years, disability was associated with uneven differences in SRH outcomes. Severe disability was strongly associated with lower knowledge of modern contraception, whereas mild disability was associated with higher odds of unintended pregnancy among currently married or cohabiting women with a recent live birth. Severe disability was also associated with higher adjusted odds of unmet need for family planning, although the estimate was imprecise and should be interpreted cautiously given the small severe-disability subgroup. Functional-domain analyses suggested that communication, hearing, and self-care difficulties were associated with the largest differences in contraceptive knowledge and unintended pregnancy. Stratified analyses suggested that disability-related differences in unintended pregnancy were larger among older women, women with less than primary education, and women in the poorest wealth quintile.

These findings add to the limited population-based evidence on SRH among women with disabilities in LMICs (10–14). They also show why disability should not be treated as a single homogeneous exposure. The pattern of results differed by severity, functional domain, and outcome: severe disability was most clearly associated with lower contraceptive knowledge, mild disability with unintended pregnancy, and communication-related difficulties with both. This pattern is consistent with systematic-review evidence that women with disabilities face barriers related to information access, communication, service delivery, provider attitudes, and financial or structural constraints (4, 8), and with qualitative evidence from rural Cambodia documenting constrained SRH information and autonomy among women with disabilities (6).

### Interpretation and comparison with previous studies

The contrast between near-universal contraceptive knowledge and elevated unintended pregnancy among women with mild disability is central to the interpretation of this study. Awareness of modern methods may be necessary, but it does not ensure access to acceptable methods, method continuation, privacy, or reproductive decision-making power. The CDHS did not directly measure counselling quality, autonomy, reproductive coercion, or provider behaviour; these mechanisms therefore could not be tested in this analysis. However, the observed pattern is compatible with prior work showing that women with disabilities may encounter barriers even after they have received basic information about contraception (4, 6).

Several pathways could plausibly contribute to this knowledge-to-outcome gap. Communication barriers may reduce the effectiveness of service delivery when services rely on spoken explanations, written materials, or group sessions that are not accessible to women with hearing, communication, cognitive, or visual difficulties (4, 6, 7). Dependence on partners, relatives, or caregivers for transport, interpretation, or daily support may reduce privacy and control over contraceptive decision-making (6). Prior studies have described how restrictive norms and assumptions about sexuality, motherhood, and decision-making capacity may undermine informed and voluntary reproductive choice for women with disabilities (4, 6). These interpretations should be considered hypothesis-generating rather than causal because the CDHS did not directly measure service-delivery quality, privacy, autonomy, accessibility, or provider behaviour.

Our finding that severe disability was associated with lower contraceptive knowledge is consistent with evidence that persons with disabilities in LMICs face barriers to family-planning information and services (4, 7, 11). A recent systematic review and meta-analysis reported lower modern contraceptive use among persons with disabilities in LMICs and identified family-planning knowledge, education, wealth, and age as important correlates (11). Our results extend this evidence by suggesting that, in Cambodia, the knowledge gap was concentrated among women with severe disability and among women with communication, hearing, or self-care difficulties.

The association between mild disability and reported unintended pregnancy differs from a multi-country DHS analysis, which found that women with disabilities were not consistently disadvantaged across reproductive health indicators and, in several countries, were less likely to report unintended pregnancy (14). This contrast may reflect differences across settings in partnership patterns, the socioeconomic patterning of disability, reproductive intentions, sexual exposure, contraceptive access, or selection into the recent-birth sample. It also reinforces the value of analysing countries and SRH outcomes separately, rather than assuming that disability-related differences will follow the same direction across settings.

Functional-domain findings further support the importance of distinguishing disability type. Communication difficulty was associated with both lower contraceptive knowledge and markedly higher unintended pregnancy, and hearing difficulty showed a similar pattern. Although the mechanisms could not be tested directly, these associations may reflect differences in access to information, counselling, privacy, and service delivery. Self-care difficulty was also associated with poorer outcomes, which may reflect greater dependence on others, reduced privacy, or difficulty reaching and navigating health facilities. These domain-specific results should be interpreted cautiously because some functional difficulties were uncommon, but they provide useful signals for service design.

### Socioeconomic patterning

Stratified analyses suggested that disability-related differences in unintended pregnancy were larger among women aged 35–49 years, women with less than primary education, and women in the poorest wealth quintile. This pattern is consistent with an intersectional understanding of health inequities, in which disability intersects with gender, age, education, poverty, and other social determinants to shape access to care and reproductive autonomy (16, 17). The practical implication is that disability-inclusive family-planning strategies should be integrated with poverty reduction, education, and rural health-access strategies. Poverty-targeted programmes that do not include disability accommodations may miss women who cannot access facilities, counselling, or information. Conversely, disability-inclusive communication alone may be insufficient if transport costs, dependence on caregivers, or financial barriers prevent women from using services. Women facing multiple disadvantages simultaneously may be most likely to be missed.

### Health-system implications

The findings suggest several priorities for SRH programmes in Cambodia. Family-planning information and service delivery should be available in accessible formats, including sign-language interpretation or video, easy-read materials, audio formats, and communication support for women with hearing, communication, cognitive, or visual difficulties (4, 6, 7, 17). Provider training should address disability-competent counselling, informed and voluntary contraceptive choice, privacy, and supported decision-making (4, 6, 8). Service delivery should also account for transport, physical accessibility, appointment systems, and the additional costs associated with disability (2–4, 8, 9).

Although the CDHS did not measure specific service-delivery barriers, the domain-specific patterns observed here are consistent with the need for accessible communication, disability-competent counselling, privacy, and supported decision-making in family-planning services. These reforms should be integrated into routine SRH systems rather than treated as separate disability-specific initiatives. Cambodia's inclusion of the WG-SS in the 2021–22 CDHS provides a baseline for monitoring SRH indicators by disability status (22–24). Future health information systems and surveys should continue to disaggregate family-planning indicators by disability severity and functional domain. Such monitoring would help assess whether policy commitments under the CRPD and SDG target 3.7 translate into improved access and outcomes for women with disabilities (1–3).

### Strengths and limitations

This study has several strengths. It used a large, nationally representative survey and internationally comparable disability measures, allowing disability-related SRH differences to be examined at the population level in Cambodia. By analysing disability severity, any disability, and functional domains, the study provides a more detailed picture than analyses based only on a binary disability indicator. The inclusion of three SRH outcomes also allowed us to distinguish between knowledge, reported need, and pregnancy intention, which are related but not interchangeable dimensions of reproductive health.

Several limitations should be considered. First, the cross-sectional design precludes causal inference. This is particularly important for unintended pregnancy because pregnancy intention referred to the most recent live birth in the five years before the survey, whereas disability was measured at the time of the survey. If disability onset occurred after the pregnancy, exposure status may have been misclassified. Pregnancy intention was also reported retrospectively and may be affected by recall bias or *post hoc* rationalisation. Longitudinal data that include information on the timing of disability onset would be needed to assess temporality between disability and unintended pregnancy more directly.

Second, the outcome-specific analytic samples limit generalisability. Unmet need for family planning and unintended pregnancy were analysed among currently married or cohabiting women, following DHS definitions (26). This restriction excludes sexually active unmarried women, who may face distinct barriers to SRH information and services. The unintended-pregnancy analysis was further restricted to women with a recent live birth, excluding women who had not given birth in the preceding five years and pregnancies ending in miscarriage, stillbirth, or induced abortion.

Third, although the WG-SS is widely used for population disability measurement, it captures functional difficulty in six basic domains and may under-identify psychosocial, intellectual, pain-related, or episodic impairments (22, 23). The small number of women with severe disability, particularly among those with a recent live birth, limited precision and prevented reliable interaction testing. Finally, the CDHS did not directly measure counselling quality, provider attitudes, reproductive coercion, decision-making autonomy, privacy, accessibility of information formats, or physical accessibility of services. Pathway interpretations should therefore be understood as hypothesis-generating. Future research incorporating longitudinal designs, disability measures that capture psychosocial, intellectual, pain-related, and episodic impairments, and qualitative or mixed-methods studies of counselling quality, autonomy, and provider behaviour would help clarify the mechanisms underlying the associations reported here.

## Conclusions

Women with disabilities in Cambodia experience SRH inequities. Severe disability was associated with lower knowledge of modern contraception, while mild disability was associated with higher unintended pregnancy among married or cohabiting women with a recent live birth. Differences were particularly apparent among women with communication, hearing, or self-care difficulties and among women facing age-, education-, or wealth-related disadvantage. These findings suggest that disability-inclusive family-planning approaches in Cambodia should consider not only information provision, but also communication access, provider competence, supported decision-making, privacy, physical accessibility, and socioeconomic barriers.

## Data Availability

Publicly available datasets were analyzed in this study. This data can be found here: The data can be accessed through The DHS Program website upon registration and approval: https://dhsprogram.com/data/.
