# Supplementary material for: Disability and sexual and reproductive health outcomes among women in Cambodia: a cross-sectional analysis of the 2021–22 demographic and health survey
Source: Front Reprod Health. 2026 Jul 31;8:1897583. doi: 10.3389/frph.2026.1897583 (PMC13472998; doi:10.3389/frph.2026.1897583)
Supplement: Supplementary file 1 [file Supplementaryfile1.docx]

**Supplementary Materials**

**Supplementary Table 1. Factors associated with knowledge of modern contraception among women with disabilities (n = 3,554)**

*OR, odds ratio; AOR, adjusted odds ratio. AORs are adjusted for age, education, marital status, household wealth, and place of residence. Estimates are obtained from survey-weighted logistic regression accounting for the complex CDHS sampling design.*

| **Variable** | **Knowledge: Yes (%)** | **Knowledge: No (%)** | **OR (95% CI)** | **p** | **AOR (95% CI)** | **p** |
| --- | --- | --- | --- | --- | --- | --- |
| ***Education*** | | | | | | |
| Less than primary (ref.) | 98.10 | 1.90 | 1 | n/a | 1 | n/a |
| Primary | 99.31 | 0.69 | 2.77 (1.15- 6.72) | 0.024 | 11.92 (3.78-37.59) | <0.001 |
| Secondary or higher | 100.00 | 0.00 | n/a | n/a | n/a | n/a |
| ***Age group, years*** | | | | | | |
| 15-24 (ref.) | 95.15 | 4.85 | 1 | n/a | 1 | n/a |
| 25-34 | 98.87 | 1.13 | 4.46 ( 1.39-14.29) | 0.012 | 1.58 ( 0.46- 5.48) | 0.466 |
| 35-49 | 99.23 | 0.77 | 6.57 ( 3.13-13.81) | <0.001 | 2.67 ( 1.06- 6.76) | 0.038 |
| ***Marital status*** |  |  |  |  |  |  |
| Never married (ref.) | 91.20 | 8.80 | 1 | n/a | 1 | n/a |
| Currently married/cohabiting | 99.84 | 0.16 | 60.07 (21.06-171.32) | <0.001 | 93.33 (30.78-283.01) | <0.001 |
| Divorced/separated | 99.57 | 0.43 | 22.21 ( 2.90-170.33) | 0.003 | 37.37 ( 4.48-312.08) | <0.001 |
| Widowed | 99.67 | 0.33 | 29.61 ( 3.98-220.08) | <0.001 | 53.19 ( 6.83-414.03) | <0.001 |
| ***Household wealth quintile*** | | | | | | |
| Poorest (ref.) | 98.38 | 1.62 | 1 | n/a | 1 | n/a |
| Poorer | 98.49 | 1.51 | 1.08 ( 0.46- 2.52) | 0.861 | 1.22 ( 0.48- 3.10) | 0.675 |
| Middle | 98.42 | 1.58 | 1.03 ( 0.31- 3.38) | 0.961 | 1.12 ( 0.43- 2.92) | 0.816 |
| Richer | 98.66 | 1.34 | 1.21 ( 0.40- 3.64) | 0.731 | 1.00 ( 0.25- 4.06) | 0.997 |
| Richest | 99.59 | 0.41 | 3.96 ( 1.13-13.93) | 0.032 | 2.74 ( 0.40-18.94) | 0.305 |
| ***Place of residence*** | | | | | | |
| Urban (ref.) | 98.81 | 1.19 | 1 | n/a | 1 | n/a |
| Rural | 98.61 | 1.39 | 0.85 ( 0.32- 2.29) | 0.752 | 1.40 ( 0.41- 4.80) | 0.592 |

**Supplementary Table 2. Factors associated with unmet need for family planning among currently married women with disabilities (n = 2,869)**

*OR, odds ratio; AOR, adjusted odds ratio. AORs are adjusted for age, education, household wealth, and place of residence. Estimates are obtained from survey-weighted logistic regression accounting for the complex CDHS sampling design.*

| **Variable** | **Unmet need: Yes (%)** | **Unmet need: No (%)** | **OR (95% CI)** | **p** | **AOR (95% CI)** | **p** |
| --- | --- | --- | --- | --- | --- | --- |
| ***Education*** | | | | | | |
| Less than primary (ref.) | 12.58 | 87.42 | 1 | n/a | 1 | n/a |
| Primary | 12.73 | 87.27 | 1.01 ( 0.77- 1.33) | 0.924 | 1.14 ( 0.86- 1.52) | 0.349 |
| Secondary or higher | 7.46 | 92.54 | 0.56 ( 0.28- 1.14) | 0.108 | 0.75 ( 0.36- 1.60) | 0.461 |
| ***Age group, years*** | | | | | | |
| 15-24 (ref.) | 15.73 | 84.27 | 1 | n/a | 1 | n/a |
| 25-34 | 11.41 | 88.59 | 0.69 ( 0.33- 1.45) | 0.325 | 0.77 ( 0.36- 1.63) | 0.492 |
| 35-49 | 12.48 | 87.52 | 0.76 ( 0.39- 1.49) | 0.428 | 0.81 ( 0.41- 1.63) | 0.559 |
| ***Household wealth quintile*** | | | | | | |
| Poorest (ref.) | 13.48 | 86.52 | 1 | n/a | 1 | n/a |
| Poorer | 13.24 | 86.76 | 0.98 ( 0.66- 1.46) | 0.919 | 1.00 ( 0.67- 1.51) | 0.981 |
| Middle | 16.97 | 83.03 | 1.31 ( 0.89- 1.92) | 0.165 | 1.37 ( 0.92- 2.03) | 0.123 |
| Richer | 9.25 | 90.75 | 0.65 ( 0.42- 1.03) | 0.067 | 0.73 ( 0.46- 1.16) | 0.184 |
| Richest | 7.93 | 92.07 | 0.55 ( 0.33- 0.92) | 0.022 | 0.68 ( 0.38- 1.22) | 0.195 |
| ***Place of residence*** | | | | | | |
| Urban (ref.) | 9.12 | 90.88 | 1 | n/a | 1 | n/a |
| Rural | 13.73 | 86.27 | 1.59 ( 1.14- 2.20) | 0.006 | 1.28 ( 0.88- 1.87) | 0.199 |

**Supplementary Table 3. Factors associated with unintended pregnancy among currently married women with disabilities who had a live birth in the previous five years (n = 636)**

*OR, odds ratio; AOR, adjusted odds ratio. AORs are adjusted for age, education, household wealth, and place of residence. Estimates are obtained from survey-weighted logistic regression accounting for the complex CDHS sampling design.*

| **Variable** | **Unintended pregnancy: Yes (%)** | **Unintended pregnancy: No (%)** | **OR (95% CI)** | **p** | **AOR (95% CI)** | **p** |
| --- | --- | --- | --- | --- | --- | --- |
| ***Education*** | | | | | | |
| Incomplete primary (ref.) | 27.53 | 72.47 | 1 | n/a | 1 | n/a |
| Primary | 19.66 | 80.34 | 0.64 ( 0.39- 1.07) | 0.090 | 1.09 ( 0.61- 1.96) | 0.773 |
| Secondary or higher | 21.64 | 78.36 | 0.73 ( 0.36- 1.47) | 0.372 | 1.88 ( 0.75- 4.69) | 0.178 |
| ***Age group, years*** | | | | | | |
| 15-24 (ref.) | 12.96 | 87.04 | 1 | n/a | 1 | n/a |
| 25-34 | 16.79 | 83.21 | 1.35 ( 0.59- 3.09) | 0.470 | 1.44 ( 0.62- 3.36) | 0.399 |
| 35-49 | 36.91 | 63.09 | 3.93 ( 1.75- 8.84) | <0.001 | 4.49 ( 1.93-10.41) | <0.001 |
| ***Household wealth quintile*** | | | | | | |
| Poorest (ref.) | 28.51 | 71.49 | 1 | n/a | 1 | n/a |
| Poorer | 25.63 | 74.37 | 0.86 ( 0.45- 1.65) | 0.658 | 0.78 ( 0.39- 1.58) | 0.495 |
| Middle | 25.34 | 74.66 | 0.85 ( 0.40- 1.79) | 0.670 | 0.78 ( 0.34- 1.78) | 0.551 |
| Richer | 18.79 | 81.21 | 0.58 ( 0.31- 1.10) | 0.095 | 0.43 ( 0.19- 0.94) | 0.035 |
| Richest | 17.50 | 82.50 | 0.53 ( 0.20- 1.43) | 0.211 | 0.34 ( 0.11- 1.05) | 0.061 |
| ***Place of residence*** | | | | | | |
| Urban (ref.) | 24.53 | 75.47 | 1 | n/a | 1 | n/a |
| Rural | 23.50 | 76.50 | 0.95 ( 0.50- 1.80) | 0.863 | 0.64 ( 0.32- 1.27) | 0.201 |
